# The Nature-Inspired Metaheuristic Method for Predicting the Creep Strain of Green Concrete Containing Ground Granulated Blast Furnace Slag

**DOI:** 10.3390/ma12020293

**Published:** 2019-01-17

**Authors:** Łukasz Sadowski, Mehdi Nikoo, Mohd Shariq, Ebrahim Joker, Sławomir Czarnecki

**Affiliations:** 1 Faculty of Civil Engineering, Wroclaw University of Science and Technology, WybrzezeWyspiańskiego 27, 50-370 Wroclaw, Poland; slawomir.czarnecki@pwr.edu.pl; 2 Young Researchers and Elite Club, Ahvaz Branch, Islamic Azad University, Ahvaz, Iran; sazeh84@yahoo.com; 3 Department of Civil Engineering, Aligarh Muslim University, Aligarh 202001, India; mshariqdce@gmail.com; 4 Department of Civil Engineering, Dariun Branch, Islamic Azad University, Dariun, Iran; joker_ebrahim@yahoo.com

**Keywords:** concrete, ground granulated blast furnace slag, creep strain, artificial neural networks, firefly algorithm

## Abstract

The aim of this study was to develop a nature-inspired metaheuristic method to predict the creep strain of green concrete containing ground granulated blast furnace slag (GGBFS) using an artificial neural network (ANN)model. The firefly algorithm (FA) was used to optimize the weights in the ANN. For this purpose, the cement content, GGBFS content, water-to-binder ratio, fine aggregate content, coarse aggregate content, slump, the compaction factor of concrete and the age after loading were used as the input parameters, and in turn, the creep strain (*ε*_cr_) of the GGBFS concrete was considered as the output parameters. To evaluate the accuracy of the FA-ANN model, it was compared with the well-known genetic algorithm (GA), imperialist competitive algorithm (ICA) and particle swarm optimization (PSO). Results indicated that the ANNs model, in which the weights were optimized by the FA, were more capable, flexible and precise than other optimization algorithms in predicting the *ε*_cr_ of GGBFS concrete.

## 1. Introduction

The time-dependent deformation of concrete, as a result of creep strains, severely affects the durability of concrete structures. As stated by El-Shafie and Aminah [1], the stochastic nature of creep deformation and its reliance on a large number of uncontrolled parameters (e.g., relative humidity, time of load application, stress level) makes the process of the prediction and development of accurate mathematical models very difficult (almost impossible). Thus, due to the great number of variable and uncertain parameters. As pointed out by Hołowaty [2], current empirically based models in design codes are too simplified, and therefore the real-time behavior of concrete structures is still not fully understood.

The creep strain (*ε*_cr_) depends primarily on the composition of the concrete. This composition has recently been more frequently modified using eco-friendly admixtures. The concrete obtained applying these kinds of admixtures are usually known as “green concrete”. One example of these admixtures is ground granulated blast furnace slag (GGBFS). GGBFS is a fine powder obtained by grinding the blast furnace by-product of the steel industry [3]. The application of GGBFS in sustainable cement based materials is usually beneficial. This is mainly due to a slower hydration rate in comparison with ordinary Portland cement, and also the decrease in the emission of CO_2_. However, there is lack of research on the influence of GGBFS at creep in literature. In some cases, the application of GGBFS in cement-based materials lowers the creep strain, whereas in others the creep strain is higher in comparison with concrete made of ordinary Portland cement [3,4]. Many prediction models have been proposed for assessing the creep of ordinary concrete [5,6,7,8,9,10]. Due to the intensive development of the application of GGBFS, these prediction models are not adequate for GGBFS based concrete. The actual prediction models are not able to reproduce the behavior of concrete, for which a high part of the cement is replaced by slag. However, new models have been developed in order to consider the influence of slag on the properties of concrete [11].

The conventional approach to calculate the *ε*_cr_ requires a series of laboratory tests to be performed. These tests are expensive and time-consuming. Thus, each possibility of a less expensive and faster prediction of the *ε*_cr_ of GGBFS concrete may be very useful for designing reinforced and pre-stressed concrete structures. This might be done based on the information concerning the composition of the concrete mixture and/or the values of the selected rheological properties. Artificial neural networks (ANNs) can be especially useful for this purpose. ANNs are artificial models of the biological connection and cooperation between the brain and the rest of the body. They very often work in a similar manner to human cells, using a one-way signal flow. The main advantage of ANNs is that they can handle a large amount of datasets. The use of ANNs is also beneficial due to their ability to detect complex relationships between independent variables. The method based on ANNs can be utilized as a supplementary tool for creep strain prediction. Thanks to the application of ANNs, the series of expensive and time-consuming tests may not be necessary.

Several researchers have recently tried to predict the *ε*_cr_ in concrete using ANN models. For example, Karthikeyan et al. [12] and Gedam et al. [13] predicted the creep of high performance concrete (HPC). Baland and Bodin [14] used the nonparametric ANN model for this purpose. However, most of the attempts were performed based on the simplest back propagation ANN model (e.g., the Widrow–Hoff algorithm in [15], Levenberg–Marquardt algorithm in [12]). Recently, the intensive development of the application of more advanced optimization algorithms in ANNs has been noted in the prediction of properties of green (eco) concrete [16,17]. It includes the use of genetic algorithms (GA), which have recently been used for predicting the compressive strength of concrete [18], the displacement of floors [19], the elastic modulus of recycled aggregate concrete [20,21] or the coefficient of safety in soil stabilization [22]. Multi-objective genetic programming has been used successfully by Gandomi et al. [23] in concrete creep formulation. The imperialist competitive algorithm (ICA), particle swarm optimization (PSO) and firefly algorithm (FA) are also getting more attention [24]. These algorithms are usually used to improve the learning processes of ANNs. Thus, in this article, the authors decided to use the FA for this purpose.

The firefly is a very interesting insect, and their spectacular plays have inspired poets and scientists. Many researchers have studied the behavior of fireflies in nature [24]. The nature-inspired FA is a metaheuristic algorithm proposed by Xin-She Yang [25] and inspired by the flashing behavior of fireflies. In the standard firefly algorithm, two important issues need to be defined. According to [24], the intensity of light is defined as:

(1) $$I\left(r\right)=I_{0}e^{-\gamma r^{2}},$$

where I_0_ represents the intensity of the source light and the absorption of light by approximating the constant coefficient of light absorption γ.

The intensity of light *I* is referred to as an absolute measure of emitted light by the firefly, while the attractiveness *β* is the measure of light seen by the other fireflies and defined by Equation (2):

(2) $$\beta =\beta_{0}e^{-\gamma r^{2}},$$

Where $\beta_{0}$= the attractiveness at the Euclidean distance *r* is defined by Equation (3) between two fireflies *s*_i_ and *s*_j,_ and is equal to 0:

(3) $$r_{ij}=\left|\left|s_{i}-s_{j}\right|\right|\sqrt{\overset{k=n}{\underset{k=1}{\sum }}{\left(s_{ik}-s_{jk}\right)}^{2}}$$

According to Equations (1) and (2), there are two asymptotic behaviors of the FA. If γ→0, the attractiveness is constant ($\beta =\beta_{0}$), and if γ→∞, the firefly movement becomes a random walk.

The FA is being more frequently used in civil engineering applications. For example, Bui et al. [26] used the FA in a modified ANN model based expert system for predicting the compressive and tensile strength of high-performance concrete. Moreover, Sheikholeslami et al. [27] used an improved FA with an upper bound strategy to optimize reinforced concrete retaining walls. Additionally, Nigdeliet al. [28] found this method useful to optimize reinforced concrete footings.

To the best of our knowledge, there is no developed method for predicting the creep strain of concrete with ground granulated blast furnace slag using the FA. Thus, this study aims to apply the FA in order to optimize the weights of an ANN model for determining the creep strain of GGBFS concrete. The obtained results were validated using the well-known GA, ICA and PSO.

## 2. Experimental Setup

### 2.1. Materials

Locally available ordinary 43 grade (C43) Portland cement (OPC) and the commercially available GGBFS were used in the experiments (Indorama cement industry, Raipur, Maharashtra, India). The physical properties are presented in Table 1, while the selected chemical properties of the cement and GGBS are given in Table 2 (IS 8112, 1989 [29] and IS 12089, 1999 [30]), respectively.

**Figure 1:**
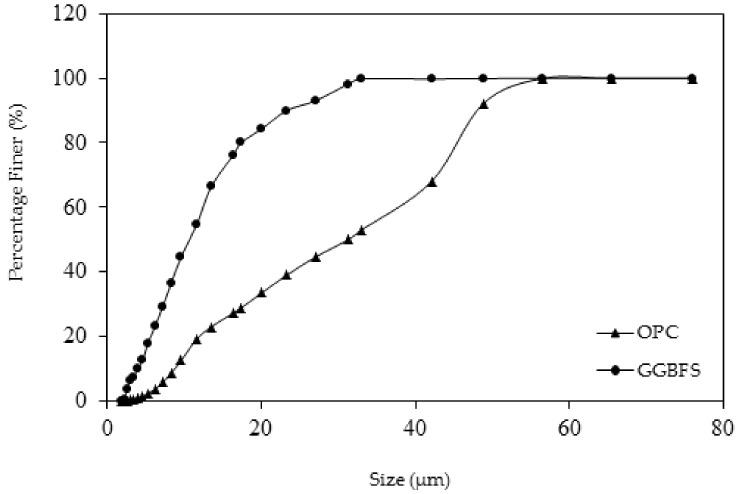
Particle size distribution of the ordinary Portland cement and ground granulated blast furnace slag.

The particle size distribution of the OPC and GGBFS was carried out using the Malvern particle size analyzer, which has Helium-Neon laser rays with a micron range of 0.5 to 560. Isopropyl alcohol with sodium pyrophosphate was used for the dispersion of particles. The cement and GGBFS were stored in airtight silos to protect them from moisture. The test results of the particle size distribution of the OPC and GGBFS are given in Figure 1.

**Figure 2:**
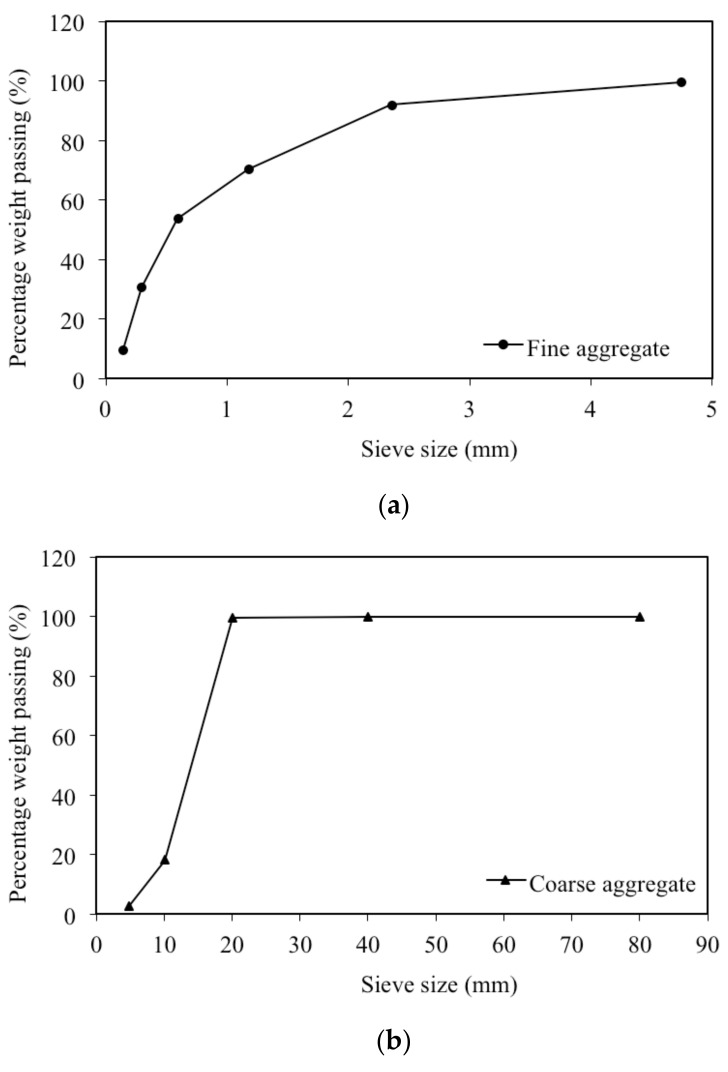
Particle size distribution of: (**a**) Fine aggregate; (**b**) Coarse aggregate.

Locally available natural river sand with a fineness modulus of 2.45 was used as the fine aggregate. The particle size distribution of the sand is given in Figure 2a. Locally available crushed stone basalt aggregate with a maximum nominal size of 16 mm and a fineness modulus of 6.8 was used as the coarse aggregate. The sieve analysis is given in Figure 2b.

Initially, based on the method of trial, three OPC concrete mixes were prepared with a target compressive strength of 45 MPa, 35 MPa and 25 MPa. Five specimens from each mix were tested at the age of 7 and 28 days for the OPC concrete mix proportioning. GGBFS concrete mixes were also prepared and had a cement replacement ratio of 20%, 40% and 60%. The water-to-binder ratio changed from 0.45 to 0.55. Thus, there were three mix groups designated as M1, M2 and M3 containing four concrete mixes in each mix group i.e., a total of twelve concrete mixes were prepared. In all the concrete mixes, the fine to coarse aggregate ratio was kept constant at 0.6 while verifying the maximum density of the combined aggregate. Table 3 shows the details of the concrete mix proportions.

### 2.2. Methods

The experiments were performed in a laboratory in order to determine the creep strain of the plain and GGBFS concrete. Creep strain can be defined as the gradual increase in strain induced by the constant sustained loading applied on the concrete specimen, and can be denoted as *ε*_cr_. The sustained loading was applied for 150 days on 150 × 600 mm cylindrical specimens to investigate the *ε*_cr_ of the GGBFS concrete. As pointed out previously by Shariq et al. [31], *ε*_cr_ can be defined as:

(4) $$\varepsilon_{cr}=\frac{{\left(\delta l_{t}\right)}_{c}}{l}$$

where $\varepsilon_{cr}$ = creep strain[μm/m];${\left(\delta l_{t}\right)}_{c}$ = length changes in time due to creep [μm]; *l* = original length[m].

**Figure 3:**
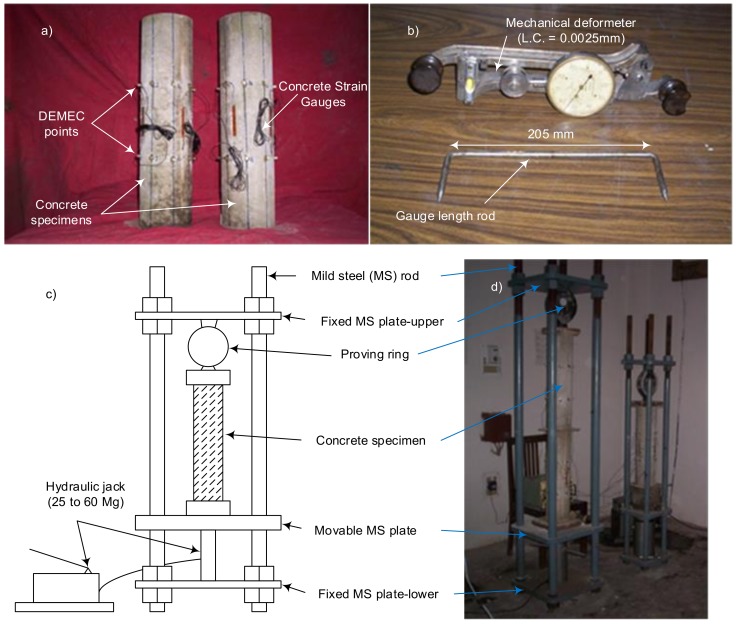
View of: (**a**) Prepared concrete specimens for creep strain measurement;(**b**)mechanical deformeter; (**c**) scheme of creep strain test setup; (**d**) creep strain specimens during the tests.

Two specimens of each mix were prepared for the creep tests. The prepared concrete specimens for the measurement of creep strain are shown in Figure 3a. For the measurement of creep, the specimen was loaded at the age of 28 days for each mix at a sustained load level of 50% of the first crack load (i.e., 20, 17 and 12 ton for M10, M20 and M30 plain concrete mixes, respectively). Before loading the creep specimens, the first crack load of the plain concrete specimen after 28 days of curing was measured on a compression testing machine, and the same load was applied for the GGBFS based concrete specimen for each mix. Hence, the stress level on the GGBFS concrete specimens was also 50% of the first crack load measured on the plain concrete specimens (i.e., 20, 17 and 12 ton for respective GGBGFS concrete mixes). A hydraulic jack was used to apply the sustained load, which was maintained through the proving ring. For each mix, the total deformation was observed at 1, 3, 7, 14, 21, 28, 30, 56, 60, 90, 120 and 150 days. The creep specimens under a sustained loading condition are shown in Figure 3b. The creep strain for a given mix was obtained as the average of the strains recorded for two specimens. All the tests were performed at an ambient temperature of 27 ± 2 °C and relative humidity of 60–65%, as recommended for laboratory conditions for Indian standards (IS 516, 1999 [32]).

On the surface of each of the creep specimen, 6 gauge lines of demountable mechanical gauge (DEMEC) points were attached at a gauge length of 205 mm in a longitudinal direction. The DEMEC gauge had countersunk holes to facilitate the mounting of a Huggenberger deformeter (Figure 3c). The size of the countersink was of at least 0.0025 mm. Four concrete strain gauges were also pasted on each of the specimens to determine the creep strain by means of a digital strain indicator (Figure 3d) in order to cross check the results. Concrete strains were measured using a 65 mm gauge length with four sets of gauge points oriented at 90° along the sides of the cylinder.

Shrinkage strain was also measured on the concrete specimen of the same size as that considered in the creep strain measurement. The shrinkage strain was recorded at the same age as the creep strain was recorded i.e., at 1, 3, 7, 14, 21, 28, 56, 90, 120, 150 and 180 days. The shrinkage strain of each mix was recorded based on the average of two specimens from each mix. Then, the creep strain was calculated as creep strain = total strain – elastic strain due to instantaneous load – shrinkage strain. The results were presented by Shariq et al. [31].

### 2.3. Statistical Analysis of the Database

The data was collected from a previous study carried out by [31,33]. Table 4 shows several tens out of 132 values of parameters obtained experimentally. Table 5 shows the statistical characteristics of the measured parameters.

In order to determine the input variables for the ANN model, or their influence on the output value, the correlation between the input variables and output variable needed to be calculated. Before that, it was necessary to choose the proper test of correlation and to know the distribution of the input variables. For this purpose, it was useful to use the Shapiro–Wilk test of compliance with normal distribution. Using this test, the values of variables are ranked in a non-significant sequence, and then the test statistic is performed. According to [34], if the probability level in this statistic is lower than the determined significance level *W*(α), the hypothesis of conformity with normal distribution should be rejected. For all the measured parameters, the Shapiro–Wilk compliance test with normal distribution was conducted according to[34]:

(5) $$W=\frac{{\left(\sum_{i=1}^{n}a_{i}x_{\left(i\right)}\right)}^{2}}{\sum_{i=1}^{n}{\left(x_{i}-\bar{x}\right)}^{2}},$$

where $x_{i}$ is the sample number, $x_{\left(i\right)}$ is the *i*_th_-smallest number in the sample and $\bar{x}$ is the sample mean. The results of the Shapiro–Wilk test for the individual parameters are shown in Table 5. In the Shapiro–Wilk test, if the level of *W* probability of this statistic drops below the determined level of the significance *W* = 0.956 of the test, the hypothesis of compliance with normal distribution is rejected. It can be concluded from Table 5 that for the predetermined level of significance α=0.01, the hypothesis regarding compliance of the distribution of all the parameters with normal distribution needed to be rejected for all of the parameters.

Thus, in order to analyze the correlation between the individual parameters and parameter *ε*_cr_, the nonparametric Spearmann’s (*ρ*_s_) and Kendall’s (*τ*) rank correlation coefficients were used. Moreover, the Fisher–Snedecor test was also conducted in order to confirm the above assumptions [35]. In this test, the *F* value at the level of significance α=0.05 should be higher than 3.91. The values of F < 3.91means that the input variable has no effect on the creep strain value *ε*_cr_. The calculation results of the *ρ*_s_ and *τ* rank correlation coefficients and the values of *F* are presented in Table 6.

Table 6 shows that correlation coefficients *ρ*_s_ and *τ* obtained the highest value for the parameters *t*, *C* and *CF*, which may indicate the major importance of these parameters in the selection of input variables for the ANN model. The negative values of correlation coefficients in the case of parameter *G* indicated a decrease in their values with an increase of the output variable *ε*_cr_. On the other hand, the results of the Fisher–Snedecor test suggested that the most significant predictor could be *t* (with the *F* value over 66). Even the lowest values of coefficients *ρ*_s_ and *τ* were obtained for *w/b*, and the *F* value for this parameter was over 27 (much more than 3.91). According to [36], it has been proven that age after loading, as well as the parameters describing the components of the concrete responsible for the value of elastic modulus *E,* have the biggest impact on creep strain *ε*_cr_. The results of the calculations of *ρ*_s_ and *τ* rank correlation coefficients and the Fisher–Snedecor test indicated the suitability of all the obtained parameters as input parameters for the ANN model. Considering the above, the input parameters for the ANN model were age after loading (*t*), cement content (*C*), GGBFS content (*G*), water-to-binder ratio (*w/b*), fine aggregate content (*Fa*), coarse aggregate content (*Ca*), slump (*S*) and compaction factor (*CF*).The creep strain *ε*_cr_ (μmm/mm) of the GGBFS concrete was used as the output parameter of this ANN model.

## 3. Numerical Analysis

### 3.1. Selection of the Optimum Structure of the ANN Model

Among the 132 data sets, 70% of the samples (92 sets) were used for training, 15% of the samples (20 sets) were selected for cross validation and the other 15% (20 sets) were used for network testing. According to [28], as well as to the guidelines advocated by Hecht-Nielson [37] and Rogers and Dowla [38], the number of nodes in the hidden layer should not be less than the value obtained from Equation (6)that was used for the maximum determination of the number of hidden layer nodes *N*_H_:

(6) $$N_{H}\leq min\left(2N_{I}+1;\frac{N^{TR}}{N_{I}+1}\right)$$

**Figure 4:**
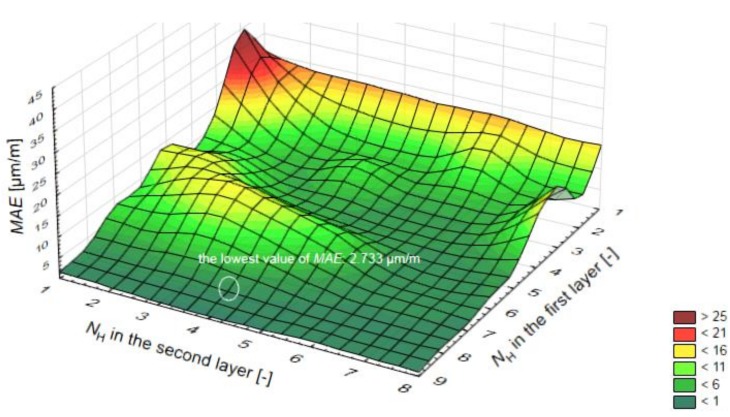
Results of mean absolute error (MAE) for different topologies of ANN models.

Where *N*_I_ is the number of input, and $N^{TR}$ is the number of training samples. Given that the number of effective inputs is equal to 8, the maximum number of nodes in the hidden layer is 17 (*N*_H_ ≤ 17), or given that the number of training samples is 92, the maximum number of nodes in the hidden layer is 10. The mean absolute error (MAE) was used for calculation and to select the most efficient topology of the ANN. The results are presented in Figure 4.

**Figure 5:**
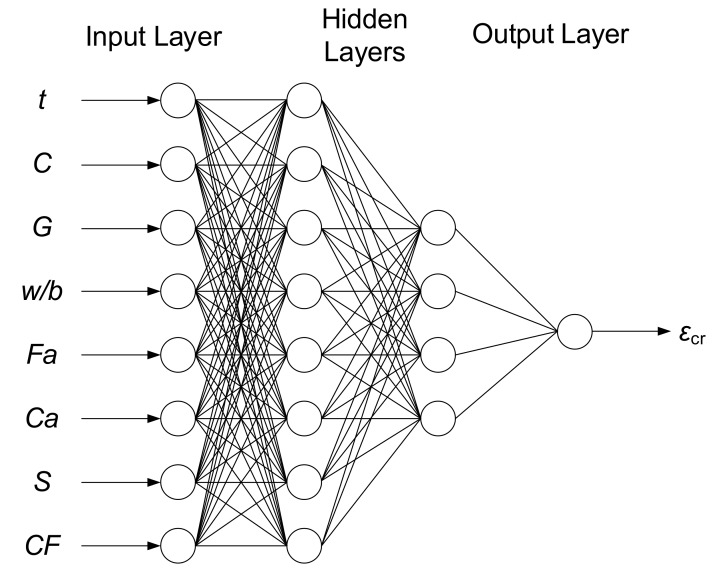
Optimum structure of the Artificial Neural Network model.

Finally, the topology of 8 input variables, 8 and 4 hidden layer nodes and 1 output variable with a MAE value of 0.80 was found to be the most accurate ANN model from all of the 72 ANN tested models. To determine the optimization of weights of each ANN model, the FA was used. In order for the models to properly function, and to determine the best one, the mean and the best criteria obtained from the models were compared with each other. According to the results, the feed forward (FF) model, in which the weights were optimized with the FA, offered the best results in the desired models through the 8-8-4-1 structure. The Tansig stimulation function and the Translim test algorithm were used in the final model. The optimum structure of the ANN model is shown in Figure 5.

**Figure 6:**
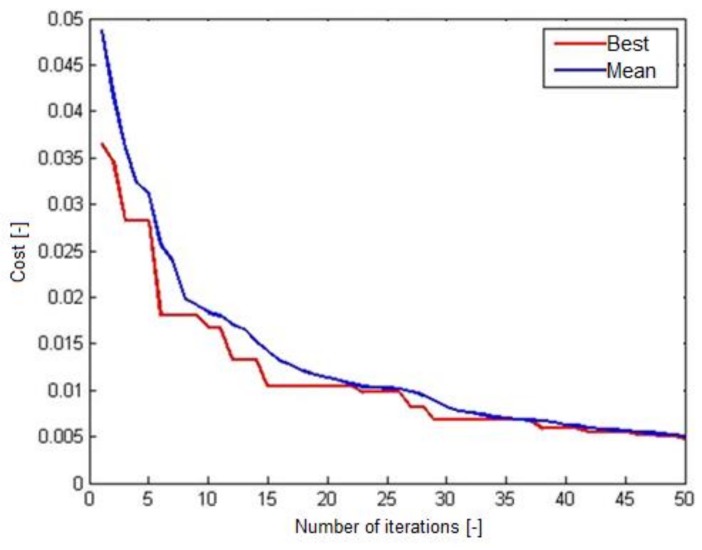
Chart in the FA-ANN with the optimum 8-8-4-1 structure.

Figure 6 shows the cost graph for 50 iterations for the FA-ANN with the optimum 8-8-4-1 structure. This graph presents the minimum and best cost in two steps. If both parameters are closer to zero by increasing the repetition, the accuracy of the model is higher. Moreover, if both parameters increase with repetition, the error rate in the model decreases.

**Figure 7:**
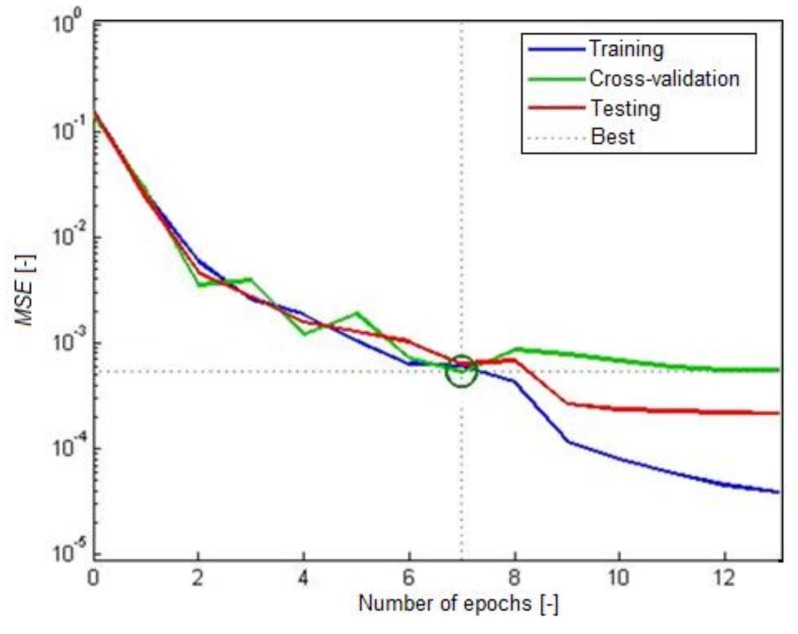
Performance in the FA-ANN with the optimum 8-8-4-1 structure.

Figure 7 shows the MSE performance in the FA-ANN with the optimum 8-8-4-1 structure for the training, cross-validation and test steps. According to this figure, the best performance was achieved at epoch 7.

### 3.2. Results of Learning, Testing and Cross-Validation of the FA-ANN

**Figure 8:**
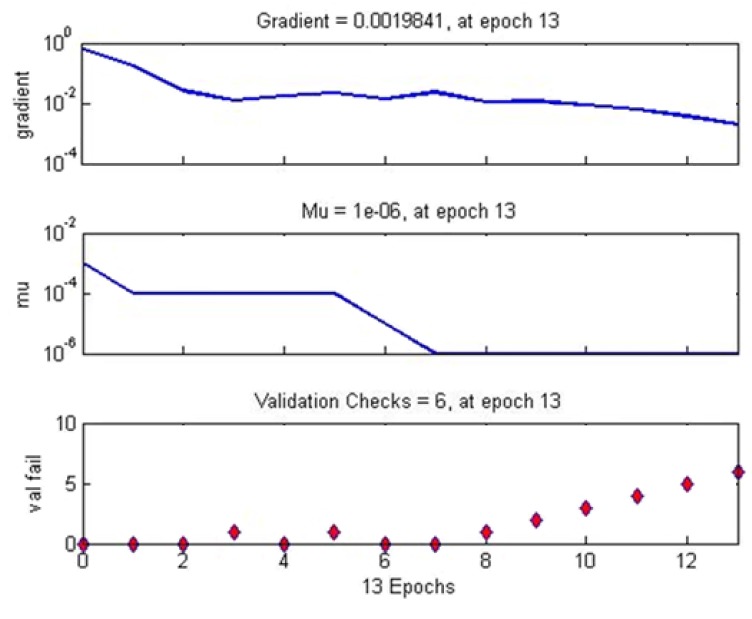
State in the FA-ANN with optimum 8-8-4-1 structure.

Figure 8 illustrates the training state of the FA-ANN. According to this figure, the errors were repeated 6 times after epoch number 7, and the acting was stopped at epoch 13. In other words, the last epoch before the error repetitions, namely epoch 7, was considered as the best performance and its related weights were assumed as the final model weights. Furthermore, the cross-validation check was equal to 6 since it was based on the number of repetitions.

**Figure 9:**
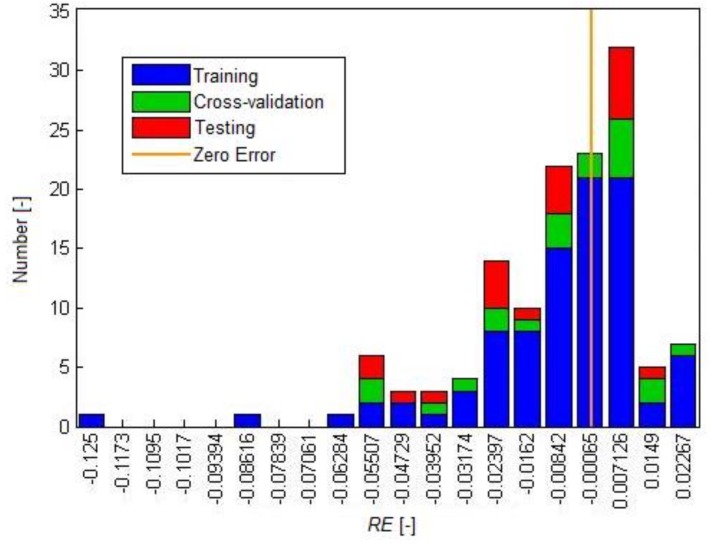
Error (*RE*) distribution at different stages of the FA-ANN with optimum 8-8-4-1 structure.

Figure 9 shows the relative error (RE) distribution histogram of the FA-ANN for the training, cross-validation and testing steps. This figure shows that the data fitting errors were distributed within a reasonably satisfactory range around zero.

### 3.3. Validation of the FA-ANN Model

In order to evaluate the accuracy of the FA-ANN model, it was compared with the other commonly used algorithms, such as the genetic algorithm (GA), imperialist competitive algorithm (ICA) and particle swarm optimization (PSO). The characteristics of these algorithms, used for validation of the FA-ANN model, are given in Table 7.

**Figure 10:**
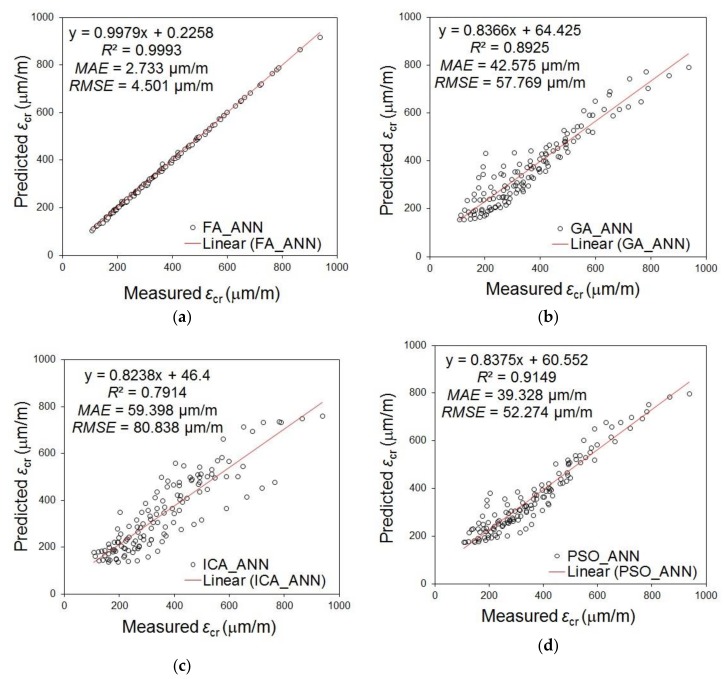
Results of experimental and computational values for the creep strain parameter using the ANN model modified by: (**a**) FA; (**b**) GA; (**c**) ICA; (**d**) PSO.

Figure 10 indicates the comparison of experimental and computational values for the creep strain parameter using the ANN model modified by the FA, ICA, GA and PSO. The results presented indicated that the ANN model optimized by the FA determined the creep strain values more accurately than other algorithms. This has been stated on the basis of the comparison of the values of linear correlation coefficient *R*, mean absolute error *MAE*, mean squared error *MSE* and root mean square error *RMSE*.

## 4. Conclusions

The conclusions drawn from the above analysis for the prediction of the creep strain of GGBFS concrete are as follows:

- It is possible to predict the creep strain of green concrete with GGBFS using artificial neural networks (ANN) and the nature-inspired metaheuristic firefly algorithm (FA).
- A reliable prediction can be conducted based on the parameters of GGBFS concrete, which characterize the composition of the concrete mixture and selected rheological properties. For this purpose, the cement content, GGBFS content, water-to-binder ratio, fine aggregate content, coarse aggregate content, slump, the compaction factor of concrete and the age after loading were used as the input parameters, and in turn the creep strain (*ε*_cr_) of GGBFS concrete was considered as the output parameter.
- The ANN model optimized by the FA was able to predict the value of *ε*_cr_ with a very high level of accuracy. The obtained values of determination coefficient (R^2^) were equal to 0.99 in training, cross-validation and testing.
- The performance of the FA-ANN was compared with other commonly used algorithms such as the imperialist competitive algorithm (ICA), genetic algorithm (GA) and particle swarm optimization (PSO). The obtained results indicated that the ANN model optimized by the FA was more accurate and provided more precision than other models.

## Figures and Tables

**Table 1:** Physical properties of the cement and ground granulated blast furnace slag (GGBFS).

Characteristic	Experimental Value	
	Cement	GGBFS
Blaine’s fineness (m^2^/kg)	245	340
Specific gravity	3.15	2.86
Soundness (mm)	1.5	1.5
Compressive strength (MPa)	45.9	40 (with 30% GGBFS)
Normal Consistency (%) OPC + 0% GGBFS OPC + 20% GGBFS OPC + 40% GGBFS OPC + 60% GGBFS	27.0 28.5 29.5 31.0	

**Table 2:** Chemical properties of the cement and ground granulated blast furnace slag (GGBFS).

Name of Oxide	Cement (%)	GGBFS
CaO SiO_2_ Al_2_O_3_ Fe_2_O_3_ MgO Na_2_O K_2_O P_2_O_5_ TiO_2_ MnO Glass content	63.71 22.18 07.35 03.82 0.95 0.28 0.11 0.05 0.27 0.04 -	38.01 37.88 14.23 0.38 9.1 0.26 0.15 0.01 0.34 0.07 91.0

**Table 3:** Concrete mix proportions.

Mix Group	Mix Designation	Cement	GGBFS	Aggregates (kg/m^3^)		Water-Binder Ratio
		(kg/m^3^)	(kg/m^3^)	Fine	Coarse	
M1	M10 M11 M12 M13	400 320 240 160	0 80 160 240	665	1107	0.45
M2	M20 M21 M22 M23	350 280 210 140	0 70 140 210	680	1132	0.50
M3	M30 M31 M32 M33	320 256 192 128	0 64 128 192	688	1145	0.55

**Table 4:** Exemplary database (based on [31,33]).

No.	Age *t* (days)	Cement Content *C*(kg/m^3^)	GGBFS Content *G*(kg/m^3^)	Water-to-Binder Ratio w/b (-)	Fine Aggregate Content *Fa* (kg/m^3^)	Coarse Aggregate Content *Ca* (kg/m^3^)	Slump *S*(mm)	Compaction Factor *CF* (-)	Creep Strain $\varepsilon_{cr}(\mu m/m)$
1	0	400	0	0.45	665	1107	41	0.9	106
2	1	400	0	0.45	665	1107	41	0.9	123
3	3	400	0	0.45	665	1107	41	0.9	145
4	7	400	0	0.45	665	1107	41	0.9	162
5	14	400	0	0.45	665	1107	41	0.9	181
6	21	400	0	0.45	665	1107	41	0.9	196
7	28	400	0	0.45	665	1107	41	0.9	204
8	56	400	0	0.45	665	1107	41	0.9	235
9	90	400	0	0.45	665	1107	41	0.9	261
…	…	…	…	…	…	…	…	…	…
132	150	128	192	0.55	688	1145	61	0.96	937

**Table 5:** Statistical characteristics of the database.

Symbol and Name of Parameter	Statistical Characteristics				Shapiro–Wilk Test Results *W*
	Mean	Minimum	Maximum	Standard Deviation	
*t* - age after loading (days)	44.54	0.00	150.00	50.26	0.796
*C* - cement content (kg/m^3^)	249.67	128.00	400.00	83.67	0.905
*G* – GGBFS content (kg/m^3^)	107.00	0.00	240.00	80.70	0.793
*w/b* – water-to-binder ratio (-)	0.50	0.45	0.55	0.04	0.769
*Fa* - fine aggregate content (kg/m^3^)	677.67	665.00	688.00	9.53	0.767
*Ca* - coarse aggregate content (kg/m^3^)	1128.00	1107.00	1145.00	15.77	0.937
*S* - slump (mm)	50.33	41.00	61.00	5.78	0.834
*CF* - compaction factor (-)	0.92	0.90	0.96	0.02	0.935
$\varepsilon_{cr}$-creep strain (μm/m)	358.98	106.00	937.00	172,55	0.937

**Table 6:** Results of Spearmann’s (*ρ*_s_) and Kendall’s (*τ*) rank correlation coefficients and values of *F.*

Symbol and Name of Parameter	*ρ*_s_	*τ*	*F*
*t* - age after loading (days)	0.597	0.453	66.34
*C* - cement content (kg/m^3^)	0.788	0.612	13.14
*G* – GGBFS content (kg/m^3^)	−0.473	−0.345	11.36
*w/b* – water-to-binder ratio (-)	0.282	0.199	27.17
*Fa* - fine aggregate content (kg/m^3^)	0.437	0.342	27.16
*Ca* - coarse aggregate content (kg/m^3^)	0.437	0.342	27.16
*S* - slump (mm)	0.437	0.342	27.25
*CF* - compaction factor (-)	0.543	0.405	26.67

**Table 7:** The characteristics of the Firefly Algorithm (FA), Genetic Algorithm (GA), imperialist competitive algorithm (ICA) and particle swarm optimization (PSO) algorithms used for validation.

*FA*		*GA*		*ICA*		*PSO*	
Attraction coefficient	0.5	Population	150	Number of initial countries	500	Swarm size	100
Mutation coefficient	0.9	Mutation rate	15	Number of initial imperialists	50		
Number of fireflies	10	Crossover rate	50	Assimilation angle coefficient(β)	2	Cognition coefficient	2
Radius reduction factor	0.95			Angle coefficient (γ)	0.5	Social coefficient	2
Generation	50	Generation	50	Generation	50	Generation	50
